# *FunFam* protein families improve residue level molecular function prediction

**DOI:** 10.1186/s12859-019-2988-x

**Published:** 2019-07-18

**Authors:** Linus Scheibenreif, Maria Littmann, Christine Orengo, Burkhard Rost

**Affiliations:** 1 Department of Informatics, Bioinformatics & Computational Biology - i12, TUM (Technical University of Munich), Boltzmannstr. 3, 85748 Garching/Munich, Germany; 2 Department of Structural and Molecular Biology, University College London, London, WC1E 6BT UK; 3 Institute for Advanced Study (TUM-IAS), Lichtenbergstr. 2a, 85748 Garching/Munich, Germany; 4 TUM School of Life Sciences Weihenstephan (WZW), Alte Akademie 8, Freising, Germany; 5 Department of Biochemistry and Molecular Biophysics & New York Consortium on Membrane Protein Structure (NYCOMPS), Columbia University, 701 West, 168th Street, New York, NY 10032 USA

**Keywords:** Protein function, Protein families, Functional families, Binding residue prediction, Protein binding sites, CATH

## Abstract

**Background:**

The CATH database provides a hierarchical classification of protein domain structures including a sub-classification of superfamilies into functional families (*FunFams*). We analyzed the similarity of binding site annotations in these *FunFams* and incorporated *FunFams* into the prediction of protein binding residues.

**Results:**

*FunFam* members agreed, on average, in 36.9 ± 0.6% of their binding residue annotations. This constituted a 6.7-fold increase over randomly grouped proteins and a 1.2-fold increase (1.1-fold on the same dataset) over proteins with the same enzymatic function (identical Enzyme Commission, EC, number). Mapping de novo binding residue prediction methods (*BindPredict-CCS, BindPredict-CC*) onto *FunFam* resulted in *consensus* predictions for those residues that were aligned and predicted alike (binding/non-binding) within a *FunFam*. This simple consensus increased the F1-score (for binding) 1.5-fold over the original prediction method. Variation of the threshold for how many proteins in the consensus prediction had to agree provided a convenient control of accuracy/precision and coverage/recall, e.g. reaching a precision as high as 60.8 ± 0.4% for a stringent threshold.

**Conclusions:**

The *FunFams* outperformed even the carefully curated EC numbers in terms of agreement of binding site residues. Additionally, we assume that our proof-of-principle through the prediction of protein binding residues will be relevant for many other solutions profiting from *FunFams* to infer functional information at the residue level.

**Electronic supplementary material:**

The online version of this article (10.1186/s12859-019-2988-x) contains supplementary material, which is available to authorized users.

## Background

Knowledge about the function of proteins is crucial for a wide array of biomedical applications. Public resources such as the Gene Ontology (GO) [1] or the Enzyme Commission (EC) classification system [2] provide hierarchical classifications of protein function (frequently also referred to as gene function). The CATH database classifies all proteins for which the three-dimensional structure (3D) is experimentally known in a hierarchy [3]. CATH has also introduced the concept of *superfamilies* linking proteins with similar 3D structures and very different sequences [4]. The largest known superfamilies are so large that the two hundred largest cover some region in 62% of known proteins [5]. Given the enormity of the span of these superfamilies, only some members of the same superfamily will function alike. Capturing those that do requires a sub-classification into functional families (called *FunFams*) [3]. CATH *FunFams* sub-classifies relatives according to similarity in their predicted specificity determining residues. COPS [6] and SCOP [7] provide two alternatives for classifying proteins according to their 3D structure, and SUPERFAMILY joins the sequence-based and the structure-based view of linking families. These classifications capture mostly taxonomical rather than functional relations [8] while using *FunFams* allows the prediction of protein functions as assessed by CAFA [9].

**Fig. 1:**
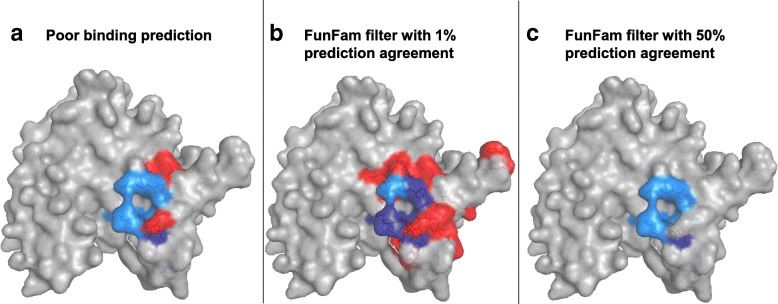
Concept of using *FunFam* to filter binding residue predictions. For the example of protein glutathione S-transferase (identifier 1U3I [17, 18]) binding glutathione. The binding residues were shown on the structure using PyMol [19]. Correctly predicted binding residues (TP) are shown in darkblue, incorrectly predicted non-binding residues (FN) in lightblue, and incorrectly predicted binding residues (FP) in red. **a** Poor binding prediction: Some prediction method (here *BindPredict-CCS*) might correctly identify only a small fraction of all binding residues (here in red with a precision = recall = F1 = 11%). The method might even incorrectly over-predict more residues as binding (red) and might miss more observed binding residues (lightblue) than it gets right. **b** *FunFam* filter with 1% prediction agreement: Simply filtering the prediction by requiring that at least 1% of all proteins aligned at a particular residue position had the same binding residue prediction (consensus threshold = 0.01). For the example, given, this boosted recall to 67% (precision = 16%, F1 = 26%). **c** *FunFam* filter with 50% prediction agreement: Filtering the prediction by requiring consensus threshold of 0.5 (50% of the residues predicted equally) removed most predicted binding residues without removing the correctly predicted ones (correctly predicted residues shown in darkblue identical in **a** and **c**; precision = 20%, recall = 11%, F1 = 14%)

One problem in assessing functional protein classifications is the following common logical circularity: classifications are based on function annotations for full-length proteins (rather than functional units such as domains) and the reliability of these classifications are measured applying the same type of annotations [10]. In the assessment of machine-learning, developers spend substantially resources to evade such a circularity through careful cross-validation and, nevertheless, fail all too often [11–13]. Since cross-validation is much less common for database annotations [14], the circularity is even more difficult to avoid. Here, we side-stepped such a vicious circle by using the similarity of binding residues between proteins as a proxy for functional similarity. Functionally similar proteins are expected to share binding residues that facilitate their common functional task, making it possible to infer similarity in overall function from similarity in binding sites. Since the annotation of protein function, e.g. through GO or EC numbers, often precedes the experimental unravelling of molecular details, our molecular proxy effectively removed the circularity thereby providing an independent means of assessing functional classifications. We added another element, namely results from two methods predicting binding residues exclusively through information available from the sequence (dubbed *BindPredict-CCS* and *BindPredict-CC* [15]). The development of the method neither used GO nor EC numbers, nor CATH nor *FunFams*, instead the most important signal originated from evolutionary couplings [16]. We hypothesized that if *FunFams* extracted relevant information about function, we would find this in the consistency of predicted binding residues within *FunFams* (more explicitly binding residues would agree more within than between *FunFams*). If true, we expected to be able to leverage the *FunFams* clustering to filter binding residue predictions as exemplified by two methods tested (Fig. 1).

## Results

### Binding residues agree for FunFams, less so for EC

After omitting all proteins without binding residue annotations (not in the PDB), those with conflicting sequence or annotation lengths, those with duplicate entries (each UniProt identifier once in each *FunFam*), and families with single members, 7,172 sequences from 1,856 *FunFams* were left. The average binding residue similarity score for these 1,856 *FunFams* was 36.9 ± 0.6% (Table 1); on average each family had 3.9 ± 0.1 proteins (Additional file 1: Figure S1). The average similarity score for randomly constructed sequence families was 5.5 ± 0.2%. Thus, the binding residue similarity within the same *FunFam* was 6.7-fold higher than that between “random families”.

**Table 1:** Average binding residue similarity for *FunFams* and EC-numbers^a^

Group	Number of families	Number of proteins	Average binding residue similarity (Eq. 1)
*Same FunFams*	1856	7172	36.9 ± 0.6
*Same EC numbers*	1080	5789	29.9 ± 0.8
*Same FunFams, EC-FunFams subset*	1103	4143	38.6 ± 0.8
*Same EC numbers, EC-FunFams subset*	833	4143	34.5 ± 0.9
*Same EC, different FunFam*	771	2893	9.6 ± 0.4
*Same FunFam, different EC*	404	2817	27.0 ± 1.0
*Same EC, same superfamily*	1006	4445	38.0 ± .0.01
*Same EC, different superfamily*	435	1155	5.22 ± 0.01

^a^*Same FunFams*: proteins within same *FunFam*; *Same EC-numbers:* proteins with identical EC number; *EC-FunFams subset:* same subset used for both similarity calculation with FunFams and within EC classes; *Same EC different FunFam:* subset of proteins with identical EC number classified into different *FunFams*; *Same FunFam different EC:* subset of proteins from same *FunFam* with different EC numbers; *Same EC, same superfamily:* proteins with identical EC number grouped into a structural superfamily; *Same EC, different superfamily*: proteins with identical EC number grouped into different superfamilies; *±*: refers to one standard error

To put the *FunFam* results into perspective of other resources, we analyzed three popular resources in the same way, namely PROSITE [20, 21], Pfam [22], and EC classes [2]. Four thousand ninety sequences in our *FunFam* dataset mapped to 588 different PROSITE patterns. The average binding residue similarity for these groups was 25.7 ± 0.8% (compared to 29.5 ± 0.8% similarity within *FunFam*s computed on the same dataset). Three thousand five hundred thirty sequences in our *FunFam* dataset mapped 656 Pfam families which had an average binding residue similarity of 26.2 ± 0.3% (compared to 30.6 ± 0.8% similarity within *FunFams* computed on the same dataset). Both approaches outperformed randomly grouped sequences more than five-fold but performed worse than FunFams (1.2-fold).

**Fig. 2:**
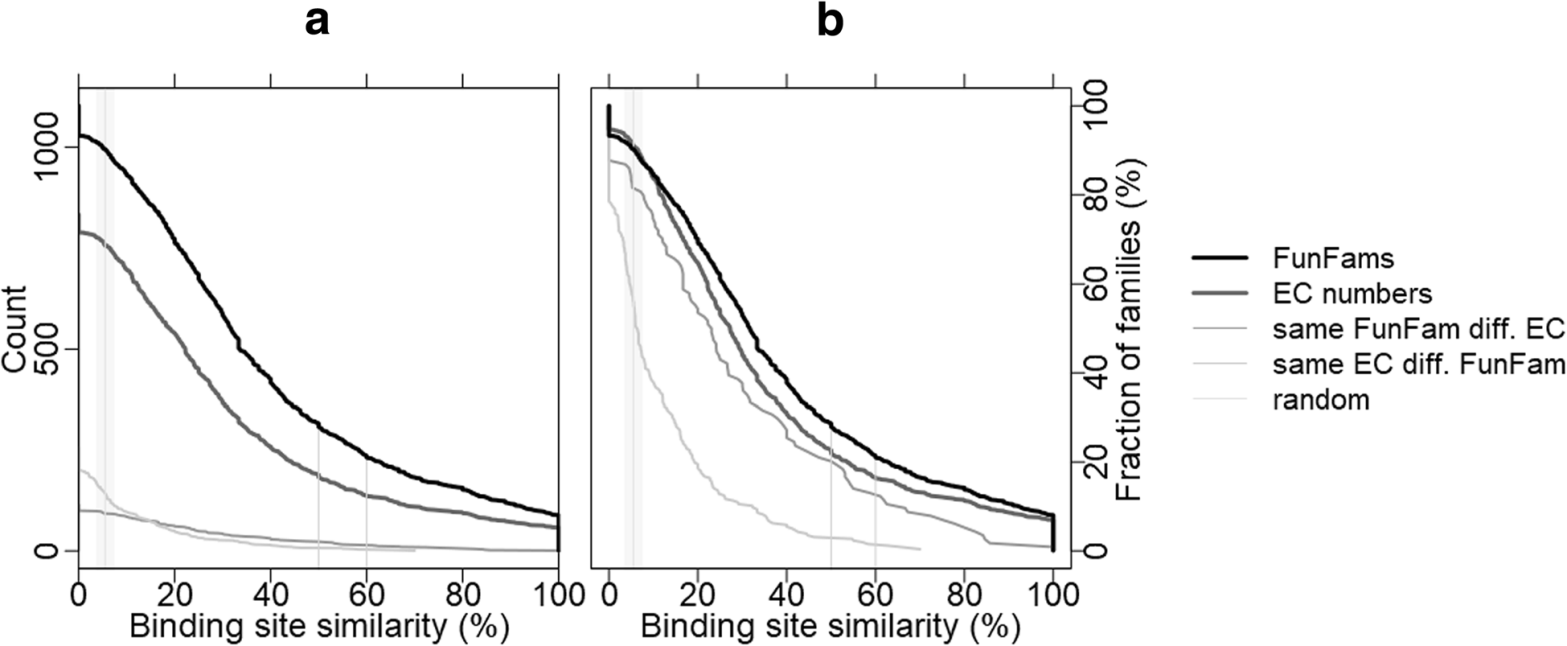
Cumulative binding residue similarities for *FunFam* and EC-number. The x-axis gives the fraction of binding residue annotations (Eq. 1) agreeing between all pairs of proteins in the same functional “groups” according to different sources: the fat black line marks the similarity within *FunFams* [3] and the gray fat line marks the similarity within same EC number [2]. For comparison the complements are also shown, namely the sub-sets of proteins in the same *FunFam* but with different EC number (dashed dark line) and in different *FunFams* but with the same EC (dashed gray line). All curves give reversely cumulative numbers answering the question: how many protein families had a binding residue annotation similarity (Eq. 1) above the similarity threshold shown on the x-axis? The two panels show the absolute count of protein families (**a**) and the fraction of all families (**b**) on the y-axis. For instance, 60% or more of all binding residues (indicated by rightmost vertical gray line; the middle vertical gray line marks the 50%) agreed within 354 *FunFams* (corresponding to 19%) and 145 identical EC numbers (corresponding to 14%). The leftmost vertical gray line marks random binding residue similarity (5.5 ± 0.2%). Contrary to all other groups, proteins grouped by the same EC number and differing *FunFams* (dashed gray line) have similarity scores close to random. The middle vertical gray lines mark the 50 and 60

For comparison with a specialized functional classification, we also computed binding residue similarity for the EC numbers classification. Our *FunFam* dataset contained 5,789 proteins with 1,080 different EC numbers (all had complete annotations for all four levels of the EC number; the remaining 1,383 proteins were ignored for this investigation). The average binding residue similarity for proteins with the same four-level EC number was 29.9 ± 0.8% (Table 1), a 5.4-fold increase over random. The binding residue similarity was higher for *FunFams* than for EC numbers across all similarity levels (Fig. 2). The average for *FunFams* was 1.2-fold higher (1.1-fold on same dataset) than for EC numbers. The same was true for particular points in the distribution, e.g. for families with 100% binding residue similarity (Fig. 2: rightmost values), and those with, e.g. 60% or 50% similarity (Fig. 2: light gray vertical lines on right and in middle). Conversely, the fraction of those with binding residue similarity levels close to random (Fig. 2: intersection of lines with gray shading on left) were higher for EC than for *FunFams*, except at zero, i.e. no binding residue similarity (*FunFams* 6.95% vs. EC numbers 6.67%).

To use the largest subsets possible, we calculated the similarity within *FunFams* and within EC classes on different subsets. To ensure that performance differences did not largely result from differences in the sub-sets, we re-computed all values for a smaller subset identical to both (4,143 proteins grouped into 1,103 *FunFams* and into 833 EC classes). On this subset, the average binding residue similarity for proteins within the same *FunFam* was 38.6 ± 0.8% that within the same EC class was 34.5 ± 0.9%, i.e. *FunFam* performed 1.1-times better than EC numbers supporting the statistically more relevant results for the larger subsets (Table 1).

We also extracted all proteins with identical EC number classified into different *FunFams* (if more than one found, one representative selected randomly). This resulted in 771 groups (each representing one EC number) with 2,893 proteins. These groups had an average binding residue similarity of 9.6 ± 0.4% (Table 1, Fig. 2: gray dashed line). Conversely, we computed the average similarity for proteins in the same *FunFam* but with different EC numbers (if several sequences in a FunFam had the same EC, we picked one at random). This yielded 404 groups (each representing one *FunFam*) with 2,817 proteins; the average binding residue similarity in this group was 26.8 ± 0.1% (Table 1, Fig. 2: dark dashed line). Along a similar line, we found that EC number annotations became more consistent when constrained by the superfamily. The average binding residue similarity for identical EC numbers rose to 38.0 ± 0.01% (1.2-fold improvement) for the subset of proteins with the same EC number and the same superfamily (with 4,445 proteins from 1006 EC numbers: Table 1). Notably, 69% of all EC numbers that occurred in a superfamily grouped into its most frequent *FunFam*. Furthermore, we found that the binding residue similarity of protein pairs with the same EC number but grouped into two different superfamilies dropped to a random level of 5.22 ± 0.01 (Table 1). The dataset contained 1,155 such proteins from 435 EC numbers.

### Binding annotation transfer within FunFams raises precision

Homology-based inference implies the following transfer: if proteins P1 and P2 are sufficiently sequence similar (e.g. PIDE(P1,P2) < T), experimental annotations obtained for P1 could be transferred to P2. We applied such a homology-based inference by transferring binding residue annotations from one member of a FunFam to all other members. This resulted in an F1 score of 37.97 ± 0.01% (Precision = 49.03 ± 0.01%, Recall = 47.52 ± 0.01%) and an MCC of 0.36 ± 0.0002. This was further evidence for the high degree of functional similarity within *FunFams*.

### Binding residue prediction improved through FunFam filter

**Fig. 3:**
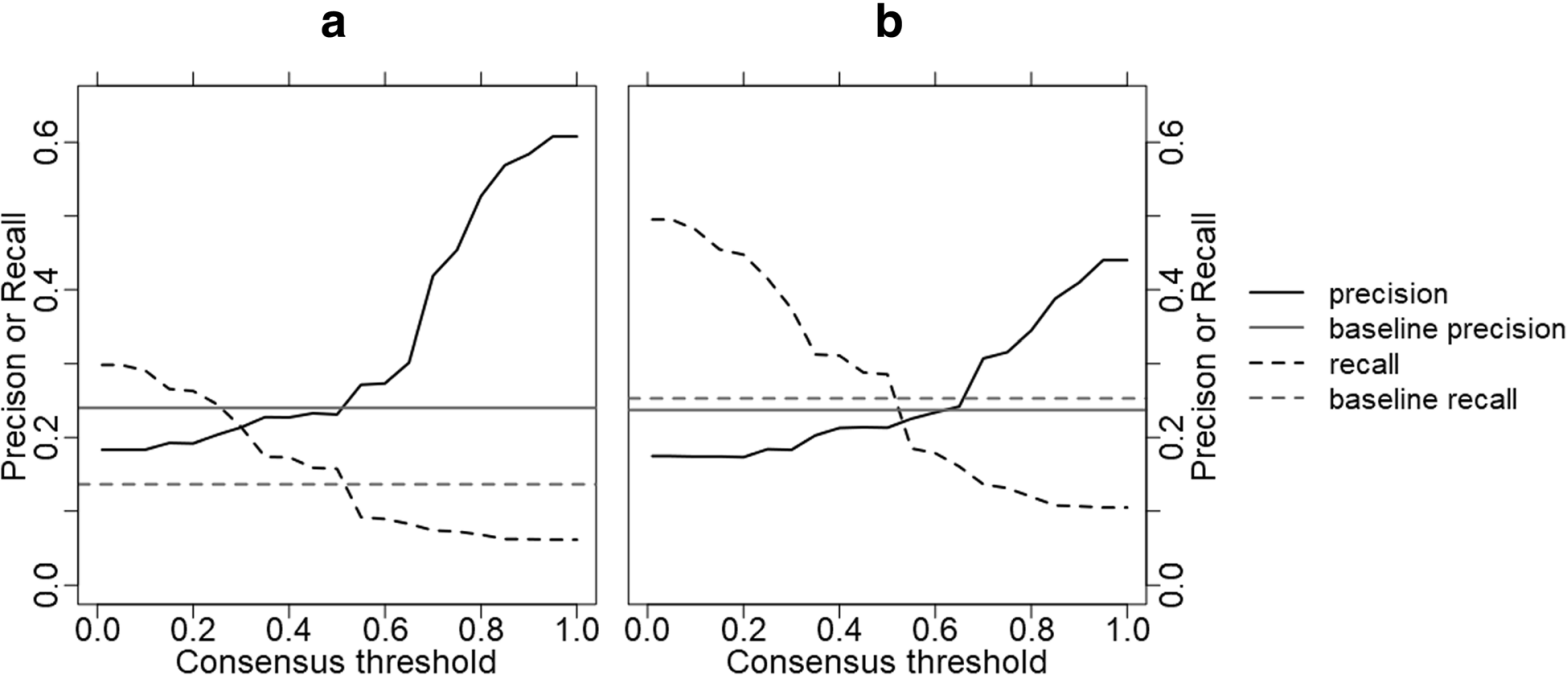
Leveraging FunFams to better predict binding residues. The horizontal lines indicate the performance estimates for precision (Eq. 2) and recall (Eq. 3) of *BindPredict-CCS* and *BindPredict-CC* baseline predictions not using *FunFams*. Predictions are refined by constructing consensus predictions for the *FunFams*. The x-axes give different thresholds in terms of what fraction of the *FunFams* members need to have a binding prediction for a particular residue in order to label that residue as binding in the consensus prediction: from at least one (0.01) to all (1.0). Depending on the threshold, both precision and recall significantly increase over the standard prediction method. The two panels illustrate the improvement over two slightly different baseline prediction methods: **a** *BindPredict-CCS* using the cumulative couplings-based input features. In this case precision increases up to 61 ± 4%. Panel **b** shows the performance improvement for *BindPredict-CC* using the clustering coefficient-based input features. For low thresholds, these predictions reach recall up to 50 ± 2%

The methods *BindPredict-CCS* and *BindPredict-CC* predict binding residues through cumulative coupling scores and clustering coefficients derived from DI scores [15]. We applied these methods to 470 proteins from 138 *FunFams*. For that set, the prediction with cumulative coupling scores reached an F1-score of 10.5 ± 1% and the prediction with clustering coefficients an F1 = 14.2 ± 1%. Building consensus predictions at consensus thresholds of 0.01 from all predictions for members of a *FunFam* raised the F1-score for cumulative coupling scores to 16.2 ± 0.8% corresponding to a 1.5-fold increase (Additional file 1: Figure S2). At the same threshold, the corresponding values for precision, recall, and accuracy were 18.3 ± 0.1% (Eq. 2), 29.8 ± 0.2% (Eq. 3) and 71.1 ± 0.1% (Eq. 4) respectively (Fig. 3a showing precision and recall). This corresponded to roughly 1.4-fold increase for precision, one-third decrease for recall and a one-tenth decrease for accuracy (data not shown). For predictions based on clustering coefficients, the F1-score increased 1.3-fold to 18.4 ± 1% (Additional file 1: Figure S2). Precision decreased 0.7-fold to 17.5 ± 1% (Eq. 2) while recall reached 49.5 ± 1% (Eq. 3), a 2.0-fold improvement (Fig. 3b). The accuracy was 55 ± 1% (1.3-fold decrease). The MCC was very low for all predictions. Nevertheless, the consensus prediction still increased the MCC about two-fold (2.1-fold for *BindPredict-CCS* at consensus threshold 0.01; 2.0-fold at 0.1; Additional file 1: Figure S3).

Varying the consensus threshold at which a binding prediction was included into the consensus, i.e. the number of proteins within a *FunFam* for which the same residue had to be predicted as binding, provided a convenient way for tuning precision and recall. At a consensus threshold of 1.0, precision reached 60.8 ± 0.4% (2.5-fold increase over standard method) for the cumulative couplings method (Fig. 3a) and 44.0 ± 0.4% (1.9-fold increase over standard method) for clustering coefficient-based predictions (Fig. 3b). At this conservation threshold, about three residues were, on average, predicted in each protein as binding and at least one residue was predicted for 55.2% of the proteins. For comparison: for the clustering coefficients, 10.4 residues were predicted as binding per protein and at least one residue was predicted for 34.4% of the proteins.

### Consensus prediction vs. machine learning prediction from bindPredictML17

To compare the consensus predictions with the results of a more sophisticated binding residue prediction method not using information from *FunFams*, we applied *bindPredictML17* [23] on 114 sequences from the *FunFam* dataset that were also part of the development set of *bindPredictML17*. For these proteins, *bindPredictML17* reached F1 = 25.85 ± 0.01% (precision = 31.40 ± 0.02%, recall = 32.59 ± 0.02%). Applying the *FunFam* filter at a consensus threshold of 0.01 led to F1 = 14.8% for *BindPredict-CC* and F1 = 19.0% for *BindPredict-CCS*. The highest recall of 43.6% was reached for *BindPredict-CC* at a consensus threshold of 0.01, and the highest precision of 50.7% for *BindPredict-CCS* for a threshold of 1.0.

## Discussion

The significantly higher binding residue similarity within the same *FunFams* than within “random families” strongly supported our hypotheses that protein binding residues proxy protein function, and that *FunFams* succeed in the classification of residue binding sites. However, the average agreement between known binding sites remained below 40%. This might be explained by five challenges. Firstly, there might be problems with *FunFams*. Secondly, too many binding sites might remain unknown. Thirdly, some experimentally annotated binding residues might not be based on cognate ligands. Fourthly, binding sites might shift without substantially affecting function. Fifthly, function might differ more between related proteins than expected. Although only the first of those five possible explanations fell within the scope of this work, we might speculate about an upper limit for the amount of the problem that could be contributed by the other four explanations. To address it, we investigated how well-known residue binding sites would agree for the popular automated resources PROSITE [20, 21] and Pfam [22], as well as, for the expert-curated EC numbers classification, considered to be the most precise existing manual curation of protein function for the subset of enzymes.

Protein families as described by PROSITE patterns or Pfam families have a clearly higher similarity in binding residue annotations than randomly grouped sequences showing that they succeed in correctly classifying proteins into families. However, the average binding residue similarity was even higher within the same *FunFam*.

On sequences grouped by their EC number, the substantial increase in binding residue similarity over random demonstrated the fine-grained classification according to catalytic function by the EC number system. Although *FunFam* classifies proteins automatically rather than driven by expert annotations, the average binding residue similarity was higher within the same *FunFam* than for identical EC numbers. Keeping the same EC number and removing the constraint “same *FunFam*” dropped binding residue similarity by about 20 percentage points (Table 1), while *FunFams* were much more robust against the removal of subsets from the same EC number (half the drop by ten percentage points, Table 1). This suggested proteins to have the same EC number only when they originated from the same *FunFam*. The binding site similarity of EC numbers constrained by CATH superfamily had a level similar to *FunFams*, but this did not imply that FunFams “only” add the superfamily classification to EC numbers as revealed by the immense drop for EC without FunFams (Fig. 2: dark dashed line). Hence, FunFams appeared to provide a more fine-grained and more consistent classification of protein function than even such a carefully expert-driven system as the EC numbers.

The *FunFam* filter managed to halve the difference in F1-score between simple prediction methods relying on only one feature and a state-of-the-art machine-learning approach. Depending on the consensus thresholds, recall or precision reached or even exceeded this approach. However, only the machine-learning approach stroke a good balance between recall and precision, therefore still outperforming the consensus prediction. This suggested that combining the consensus prediction with a more sophisticated binding residue prediction method might yield further improvements from the *FunFams* filter. We expect this expectation to be limited by the accuracy of the prediction method. In this analysis, we have focused exclusively on predictions available for all proteins of know sequences even for those that do not map to *FunFams*. Methods based on 3D structure are known to perform much better predictions, and only those can actually aspire to predict binding sites rather than binding residues [24–28].

Binding residue predictions were significantly improved using our *FunFam* filter. Besides an increase in F1-score, high consensus thresholds yielded high precision. This suggested that all proteins within a *FunFam* share some binding residues. These might be the key residues most important to maintain function and they can be identified by applying this consensus prediction (Fig. 1c). Lowering the consensus threshold increased recall. This might help to identify unknown binding residues that might be experimentally annotated in only a few *FunFam* members but might remain to be discovered in others (Fig. 1b). Overall, the fact that predictions agreed within *FunFams* constituted another, independent way to shine light on the degree to which the sub-classification of CATH super-families through *FunFam* succeeded in automatically classifying functional families. *FunFams* clearly encode functional information in the form of shared binding residues. This information was, indeed, so consistent that, e.g. binding residue predictions were improved by tapping directly into this information.

In our analysis, we focused on a few measures for the agreement of binding residue annotations and the gain in prediction performance in order to reduce the complexity of the results. As additional measures, we also applied MCC (Matthew correlation coefficient), Accuracy (or Q2, i.e. percentage of residues correctly predicted/identically annotated in either of the two states binding/non-binding), along with values for different thresholds. None of those measures changed our findings in any relevant relative way (values nominally changed but not in terms of their relative differences). We did not assess scores summarizing the entire distribution of measures such as the ROC curve or the AUC, because we can only calculate those for the baseline predictions from *BindPredict-CCS* and *BindPredict-CC* but not for the consensus prediction. The consensus prediction only provides binary labels (binding/non-binding) and lacks probabilities or prediction scores needed to compute a ROC curve. Furthermore, ROC curves using the consensus cut-off as threshold are not meaningful since this cut-off does not control the prediction outcome directly and a maximal cut-off of 1.0 does not necessarily yield false/true positive rates of 1.

The highest level of improvement in prediction performance through *FunFams* was about 0.6 (Fig. 3a: top right level of dark line marking precision). We might speculate that this suggested an upper limit for the problem of all the above five challenges (missing annotations, non-cognate ligands, shifts in binding sites neutral with respect to function and changes of function between related proteins): at most those issues matter for 40% of all binding residues, i.e. the glass is already more than half full.

## Conclusions

*FunFams* derived from CATH super-families aim at grouping functionally related proteins. Here, we showed that known binding residues are over six times (6.7) more consistent (Eq. 1) between sequences of the same *FunFam* than between sequences of different *FunFams*. *FunFams* automatically classify all proteins, nevertheless, they covered binding residue similarity about 20% better (1.2-fold increase, 1.1-fold on same dataset) than the expert curated EC numbers (Enzyme Classification) identical on all four digits for the particular classification of enzymes and about 20% (1.2-fold increase) better than PROSITE patterns or Pfam families. On top, the high similarity of binding residues for proteins with the same EC number mostly originated from the same *FunFam*. These results clearly demonstrated *FunFams* to capture functional information at the residue level with a degree of detail not matched by the EC numbers. This information was so helpful that it directly improved binding residue prediction based on evolutionary couplings (*BindPredict-CCS, BindPredict-CC*). A simple consensus prediction over many *FunFam* members yielded a substantially (30–50%) improved F1 score of 0.184 ± 0.009. Tuning the consensus threshold adjusted the precision/recall: for the highest possible threshold (1.0, meaning all members of the *FunFam* have to have that particular residue predicted as binding) precision reached as high as 60% (60.8 ± 0.4%). At this threshold, at least one binding residue was predicted for every other protein (55%). The major power of this simple analysis might lie in its generality: there was no reason why not any prediction method will improve by implementing the same filtering step.

## Methods

### Data set

The *FunFam* dataset is available online through the CATH database [3, 29]. Protein domain sequences from the same CATH superfamily are sub-classified into different *FunFams* by first performing profile-profile based comparisons between clusters of closely related sequences and applying an iterative, agglomerative clustering protocol to progressively merge clusters having profile-profile scores above a threshold. This creates a tree of putative functional relationships between clusters which is then cut by identifying differences in conserved specificity determining residues, and other likely ‘functional determinants’, between clusters. FunFams have been demonstrated to be much more structurally and functionally coherent than CATH superfamilies [3]. The *FunFam* dataset used here consisted of 1,267,077 protein domain sequences from 14,928 *FunFams*. Since *FunFams* are based on domains, there can be multiple *FunFam* assignments for the same protein. Binding site annotations were available for 7,172 proteins from 1,856 *FunFams*.

### Binding residue annotations

Binding residue annotations for sequences were obtained from the Protein Data Bank (PDB; information taken from SITE records including the description “binding site”) [17]. PDB structures were mapped to UniProt sequences through SIFT [30]. Note: we only used labels for individual binding residues without attempting to group this 1D information into 3D binding sites.

### PROSITE

PROSITE [20, 21] is a database of biologically meaningful patterns. These patterns are derived from multiple sequence alignments (MSAs) of related sequences even when the relationship is too distant to be identified solely by pairwise sequence comparisons. PROSITE patterns typically span 10–20 residues that are assumed to be important for the function of all proteins containing this pattern [21].

### Pfam families

Pfam [22] is a hidden Markov model profile base database of protein families. It provides multiple sequence alignments of protein sequences and classifies entries into the types *family, domain, motif, repeat, coiled coil* or *disordered*. Pfam strives for high quality and completeness using a highly automated procedure [31].

### EC numbers

EC numbers classify enzymes through a four-level hierarchy [2]. For example, enzymes classified as EC: 1.1.1.- are oxidoreductases (first level), acting on the CH-OH group of electron donors (second level), with NAD+ or NADP+ as an electron acceptor (third level). EC numbers might constitute the most reliable annotation of protein function despite some limits [32].

### Binding residue similarity

Binding residue annotations were compared between proteins through a simple similarity measure (Eq. 1), namely the sum over all binding residues annotated between two aligned sequences normalized by the maximum number of binding residues in one of the two.

1 $$ pairwise\_ similarity\left({X}^n,{Y}^n\right)=\frac{100}{n}\sum \limits_{i=1}^n{z}_i\kern0.5em with\ {z}_i=\left\{\begin{array}{c}1, if\ {x}_i\in \left({y}_1,\dots, {y}_m\right)\\ {}0, otherwise\end{array}\mathrm{and}\ \mathrm{n}\ge \mathrm{m}\ \mathrm{w}.\mathrm{l}.\mathrm{o}.\mathrm{g}.\right. $$

X and Y are vectors containing the indices of binding residues mapped to an alignment of the corresponding sequences. This measure was generalized to comparisons of M proteins (*M* > 2) by averaging over all M*(M-1)/2 pairwise similarities.

### Random binding residue similarity

The random similarity score was constructed as the average similarity score of randomly chosen sequences grouped into “random families”. Size and number of the “random families” was chosen to mimic the structure of the *FunFam* dataset. ClustalW aligned these randomly selected sequences [33] providing the MSA to compute the *random binding residue similarity*.

### Homology-based inference within one *FunFam*

To assess the similarity of binding residue annotations within *FunFams* we adopted a simple approach toward homology-based inference: The binding residue annotation of one *FunFam* member P1 was transferred to all other members and evaluated in comparison to the original annotation of P1. This was done in an iterative procedure such that ultimately the annotation was transferred and evaluated for each member for which it was available.

### Binding residue prediction

In this work, we focus on two basic methods derived from *bindPredictML17* [23]: *BindPredict-CCS* and *BindPredict-CC*, which are based on cumulative coupling scores (CCS) and clustering coefficients (CC) computed from evolutionary couplings [15, 23]. The evolutionary couplings were obtained by applying three publicly available tools, namely EVcouplings [34] using *jackhmmer* [35] to build families and *Freecontact* [36] to infer DI (Direct Information) scores through mean-field direct coupling analysis (more details published elsewhere [15, 23]) from these MSAs.

### Consensus prediction

The consensus prediction for a *FunFam* was built by combining the predicted labels (binding/non-binding) of residues from all sequences in the *FunFam* such that there was a prediction for each column in the MSA. A column in the MSA was predicted as binding if the fraction of sequences for which that residue was predicted as binding exceeded a *consensus threshold*. The consensus can be chosen variable to optimize precision, recall, or F1 score depending on the application. The higher the threshold, the fewer residues were predicted as binding. For instance, a consensus threshold of 0.3 for a particular residue implied that 30% of all proteins aligning at that residue position (i.e. those without insertions or deletions at that position) predicted this residue as binding.

### Performance measures

For simplicity, we used only the following standard measures to measure the success of the consensus prediction. With the standard labels TP (true positives: correctly predicted binding residues), TN (true negatives: correctly predicted as non-binding), FP (false positives: predicted as binding not observed experimentally; note that many of these constitute missing annotations, i.e. will turn into TP with greater experimental coverage), and FN (false negatives: predicted as non-binding, observed experimentally to bind). We used:

2 $$ Precision=100\bullet \frac{TP}{TP+ FP} $$

3 $$ Recall=100\bullet \frac{TP}{TP+ FN} $$

4 $$ Accuracy=100\bullet \frac{TP+ TN}{TP+ TN+ FN+ FP} $$

5 $$ F1=2\bullet \frac{Precision\ast Recall}{Precision+ Recall} $$

6 $$ MCC=100\bullet \frac{TP\bullet TN- FP\bullet FN}{\sqrt{\left( TP+ FP\right)\left( TP+ FN\right)\left( TN+ FP\right)+\left( TN+ FN\right)}} $$

All results were stated with their corresponding standard error. The standard error was calculated as standard deviation divided by the square root of *n-1. n* is the number of proteins and the standard deviation is obtained from the distribution of performances per protein.

## Additional file

Additional file 1: Supporting Online Material containing additional figures. (DOCX 5332 kb)

## Data Availability

The datasets generated and analyzed during the current study are available in the GitHub repository https://github.com/Rostlab/FunFamsConsensus. The *FunFam* dataset is available through the CATH database, http://cathdb.info.

## Abbreviations

1D: One-dimensional – information representable in a string such as “binding residues”
3D structure: Three-dimensional coordinates of protein structure
CATH: Class Architecture Topology Homology
CC: Clustering coefficients
CCS: Cumulative coupling scores
EC: Enzyme Commission (number capturing the hierarchy of enzyme function)
FunFam: Functional protein family
GO: Gene Ontology (functional annotation of proteins)
MSA: Multiple sequence alignment
PDB: Protein Data Bank

## References

[1] Ashburner M, Ball CA, Blake JA, Botstein D, Butler H, Cherry JM, Davis AP, Dolinski K, Dwight SS, Eppig JT (2000). Gene ontology: tool for the unification of biology. Nat Genet.

[2] Bairoch A (2000). The ENZYME database in 2000. Nucleic Acids Res.

[3] Sillitoe I, Cuff AL, Dessailly BH, Dawson NL, Furnham N, Lee D, Lees JG, Lewis TE, Studer RA, Rentzsch R (2012). New functional families (FunFams) in CATH to improve the mapping of conserved functional sites to 3D structures. Nucleic Acids Res.

[4] Orengo CA, Michie AD, Jones S, Jones DT, Swindells MB, Thornton JM (1997). CATH - a hierarchic classification of protein domain structures. Structure.

[5] Dessailly BH, Nair R, Jaroszewski L, Fajardo JE, Kouranov A, Lee D, Fiser A, Godzik A, Rost B, Orengo C (2009). PSI-2: structural genomics to cover protein domain family space. Structure.

[6] Suhrer SJ, Wiederstein M, Gruber M, Sippl MJ (2009). COPS - a novel workbench for explorations in fold space. Nucleic Acids Res.

[7] Murzin AG, Brenner SE, Hubbard T, Chothia C (1995). SCOP: a structural classification of proteins database for the investigation of sequences and structures. J Mol Biol.

[8] Pethica RB, Levitt M, Gough J (2012). Evolutionarily consistent families in SCOP: sequence, structure and function. BMC Struct Biol.

[9] Zhou N, Jiang Y, Bergquist TR, Lee AJ, Kacsoh BZ, Crocker AW, Lewis KA, Georghiou G, Nguyen HN, Hamid MN, et al. The CAFA challenge reports improved protein function prediction and new functional annotations for hundreds of genes through experimental screens. bioRxiv. 2019:653105. 10.1101/653105. 10.1186/s13059-019-1835-8 PMC6864930 31744546

[10] Sillitoe I, Cuff AL, Dessailly BH, Dawson NL, Furnham N, Lee D, Lees JG, Lewis TE, Studer RA, Rentzsch R (2013). New functional families (FunFams) in CATH to improve the mapping of conserved functional sites to 3D structures. Nucleic Acids Res.

[11] Moult J, Fidelis K, Kryshtafovych A, Rost B, Tramontano A (2009). Critical assessment of methods of protein structure prediction-round VIII. Proteins.

[12] Rost B, Eyrich V (2001). EVA: large-scale analysis of secondary structure prediction. Proteins Struct Funct Genet.

[13] Hamp Tobias, Rost Burkhard (2015). More challenges for machine-learning protein interactions. Bioinformatics.

[14] Mitchell Alex L, Attwood Teresa K, Babbitt Patricia C, Blum Matthias, Bork Peer, Bridge Alan, Brown Shoshana D, Chang Hsin-Yu, El-Gebali Sara, Fraser Matthew I, Gough Julian, Haft David R, Huang Hongzhan, Letunic Ivica, Lopez Rodrigo, Luciani Aurélien, Madeira Fabio, Marchler-Bauer Aron, Mi Huaiyu, Natale Darren A, Necci Marco, Nuka Gift, Orengo Christine, Pandurangan Arun P, Paysan-Lafosse Typhaine, Pesseat Sebastien, Potter Simon C, Qureshi Matloob A, Rawlings Neil D, Redaschi Nicole, Richardson Lorna J, Rivoire Catherine, Salazar Gustavo A, Sangrador-Vegas Amaia, Sigrist Christian J A, Sillitoe Ian, Sutton Granger G, Thanki Narmada, Thomas Paul D, Tosatto Silvio C E, Yong Siew-Yit, Finn Robert D (2018). InterPro in 2019: improving coverage, classification and access to protein sequence annotations. Nucleic Acids Research.

[15] Schelling M (2017). Predicting protein binding sites through machine learning with evolutionary couplings. Master’s thesis.

[16] Hopf TA, Colwell LJ, Sheridan R, Rost B, Sander C, Marks DS (2012). Three-dimensional structures of membrane proteins from genomic sequencing. Cell.

[17] Berman HM, Westbrook J, Feng Z, Gilliland G, Bhat TN, Weissig H, Shindyalov IN, Bourne PE (2000). The Protein Data Bank. Nucleic Acids Res.

[18] Chomilier J, Vaney M-C, Labesse G, Trottein F, Capron A, Mormon J-P. The crystal structure of gluthatione S-transferase from *Schistosoma mansoni*. https://www.rcsb.org/pages/policies#References.

[19] Schrodinger L (2015). The PyMOL Molecular Graphics System, Version 1.8.

[20] Sigrist CJ, de Castro E, Cerutti L, Cuche BA, Hulo N, Bridge A, Bougueleret L, Xenarios I (2013). New and continuing developments at PROSITE. Nucleic Acids Res.

[21] Sigrist CJ, Cerutti L, Hulo N, Gattiker A, Falquet L, Pagni M, Bairoch A, Bucher P (2002). PROSITE: a documented database using patterns and profiles as motif descriptors. Brief Bioinform.

[22] El-Gebali Sara, Mistry Jaina, Bateman Alex, Eddy Sean R, Luciani Aurélien, Potter Simon C, Qureshi Matloob, Richardson Lorna J, Salazar Gustavo A, Smart Alfredo, Sonnhammer Erik L L, Hirsh Layla, Paladin Lisanna, Piovesan Damiano, Tosatto Silvio C E, Finn Robert D (2018). The Pfam protein families database in 2019. Nucleic Acids Research.

[23] Schelling Maria, Hopf Thomas A., Rost Burkhard (2018). Evolutionary couplings and sequence variation effect predict protein binding sites. Proteins: Structure, Function, and Bioinformatics.

[24] Aloy P, Russell RB (2003). Understanding and predicting protein assemblies with 3D structures. Comp Funct Genomics.

[25] Betts MJ, Wichmann O, Utz M, Andre T, Petsalaki E, Minguez P, Parca L, Roth FP, Gavin AC, Bork P (2017). Systematic identification of phosphorylation-mediated protein interaction switches. PLoS Comput Biol.

[26] Duran-Frigola M, Siragusa L, Ruppin E, Barril X, Cruciani G, Aloy P (2017). Detecting similar binding pockets to enable systems polypharmacology. PLoS Comput Biol.

[27] Lewis TE, Sillitoe I, Andreeva A, Blundell TL, Buchan DW, Chothia C, Cozzetto D, Dana JM, Filippis I, Gough J (2015). Genome3D: exploiting structure to help users understand their sequences. Nucleic Acids Res.

[28] Wass MN, Kelley LA, Sternberg MJ (2010). 3DLigandSite: predicting ligand-binding sites using similar structures. Nucleic Acids Res.

[29] Dawson NL, Lewis TE, Das S, Lees JG, Lee D, Ashford P, Orengo CA, Sillitoe I (2017). CATH: an expanded resource to predict protein function through structure and sequence. Nucleic Acids Res.

[30] Velankar S, Dana JM, Jacobsen J, van Ginkel G, Gane PJ, Luo J, Oldfield TJ, O’donovan C, Martin M-J, Kleywegt G (2012). SIFTS: structure integration with function, taxonomy and sequences resource. Nucleic Acids Res.

[31] EL Sonnhammer SE, R. (1997). Durbin: Pfam: a comprehensive database of protein domain families based on seed alignments. Proteins.

[32] Mahlich Y, Steinegger M, Rost B, Bromberg Y (2018). HFSP: high speed homology-driven function annotation of proteins. Bioinformatics.

[33] Larkin MA, Blackshields G, Brown N, Chenna R, McGettigan PA, McWilliam H, Valentin F, Wallace IM, Wilm A, Lopez R (2007). Clustal W and Clustal X version 2.0. Bioinformatics.

[34] Hopf TA, Schärfe CP, Rodrigues JP, Green AG, Kohlbacher O, Sander C, Bonvin AM, Marks DS (2014). Sequence co-evolution gives 3D contacts and structures of protein complexes. eLife.

[35] Finn RD, Clements J, Arndt W, Miller BL, Wheeler TJ, Schreiber F, Bateman A, Eddy SR (2015). HMMER web server: 2015 update. Nucleic Acids Res.

[36] Kaján L, Hopf TA, Kalaš M, Marks DS, Rost B (2014). FreeContact: fast and free software for protein contact prediction from residue co-evolution. BMC Bioinformatics.

