# Supplementary material for: FunFam protein families improve residue level molecular function prediction
Source: BMC Bioinformatics. 2019 Jul 18;20:400. doi: 10.1186/s12859-019-2988-x (PMC6639920; doi:10.1186/s12859-019-2988-x)
Supplement: Supplementary file 1 — Supporting Online Material containing additional figures. (DOCX 5332 kb) [file 12859_2019_2988_MOESM1_ESM.docx]

Supporting online material
for:
Functional protein families contain (functional) information at the residue level

Linus Scheibenreif, Maria Littmann, Christine Orengo & Burkhard Rost

# Table of Contents for Supporting Online Material

[Table of Contents for Supporting Online Material 1](#_Toc12137154)

[Short description of Supporting Online Material 1](#_Toc12137155)

[Fig. S1: 2](#_Toc12137156)

[Fig. S2: 3](#_Toc12137157)

[Fig. S3: 4](#_Toc12137158)

#

# Short description of Supporting Online Material

1. Figure showing a histogram of the distribution of FunFam sizes
2. Figure showing the reverse cumulative distribution of F1 scores for a consensus prediction based on either cumulative coupling scores or clustering coefficients
3. Figure showing the change in MCC at different consensus cut-offs.

### Fig. S1:


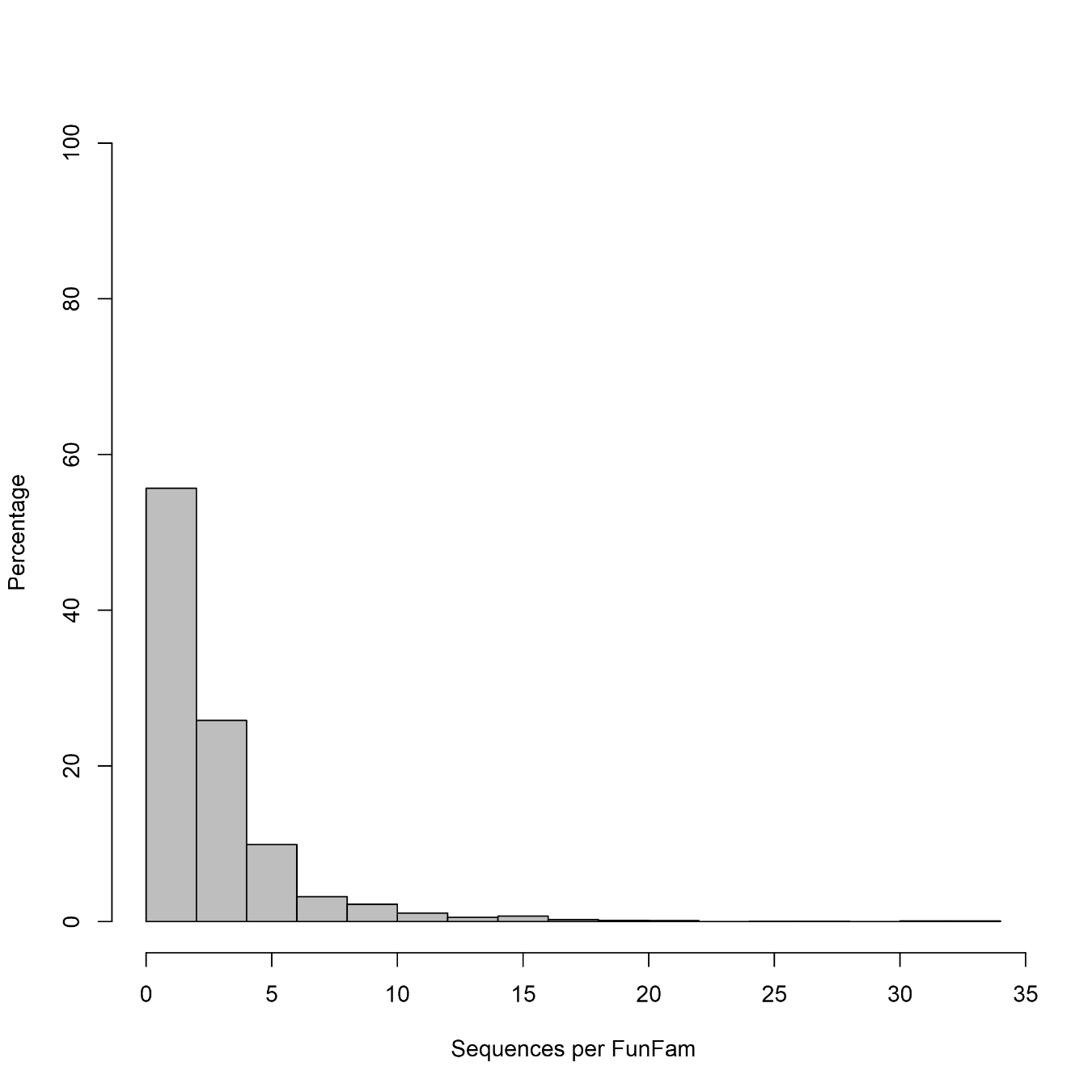


**Distribution of FunFam sizes.** Histogram plot of the number of sequences with binding site annotation per FunFam. The majority of FunFams have either 1 (21.85%) or 2 (33.81%) sequences. Only 4 (1.68%) FunFams have more than 30 sequences.

### Fig. S2:


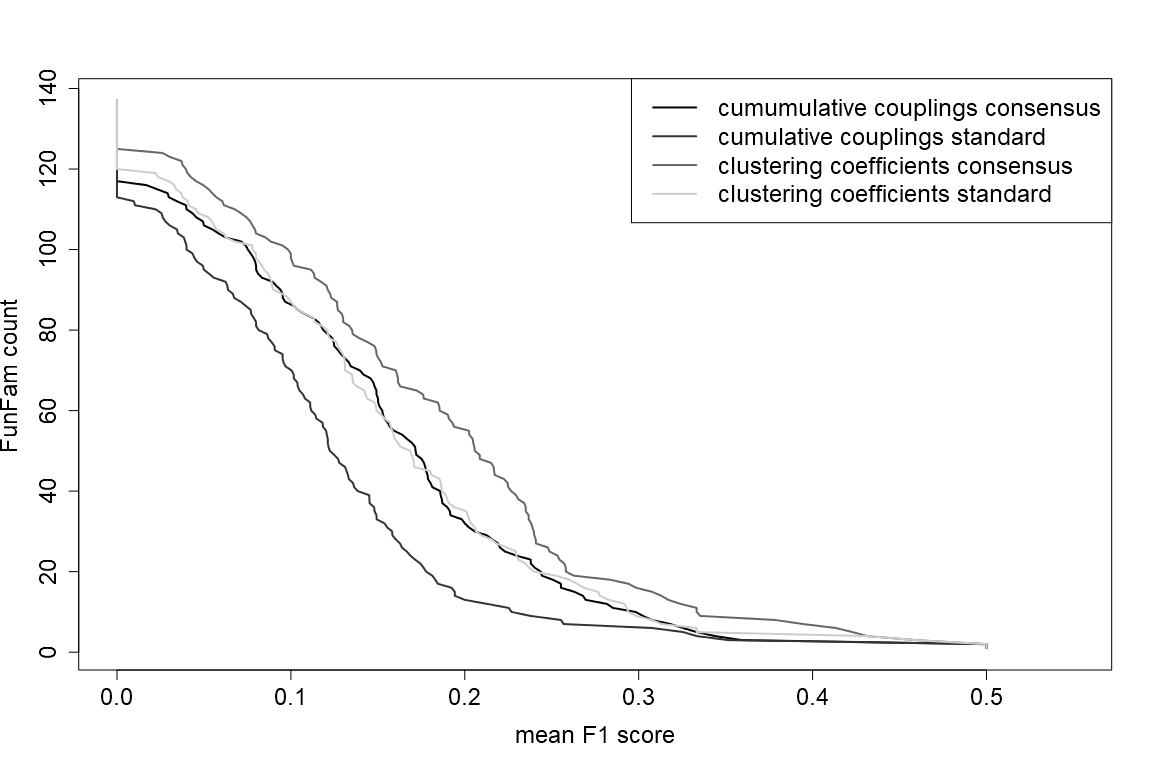


**F1 scores for consensus prediction.** Reverse cumulative plot of mean F1 scores per FunFam for the consensus prediction and the standard prediction without consensus building. The consensus approach manages to achieve significantly higher mean F1 score than the standard for both cumulative couplings- and clustering coefficients-based methods.

### Fig. S3:


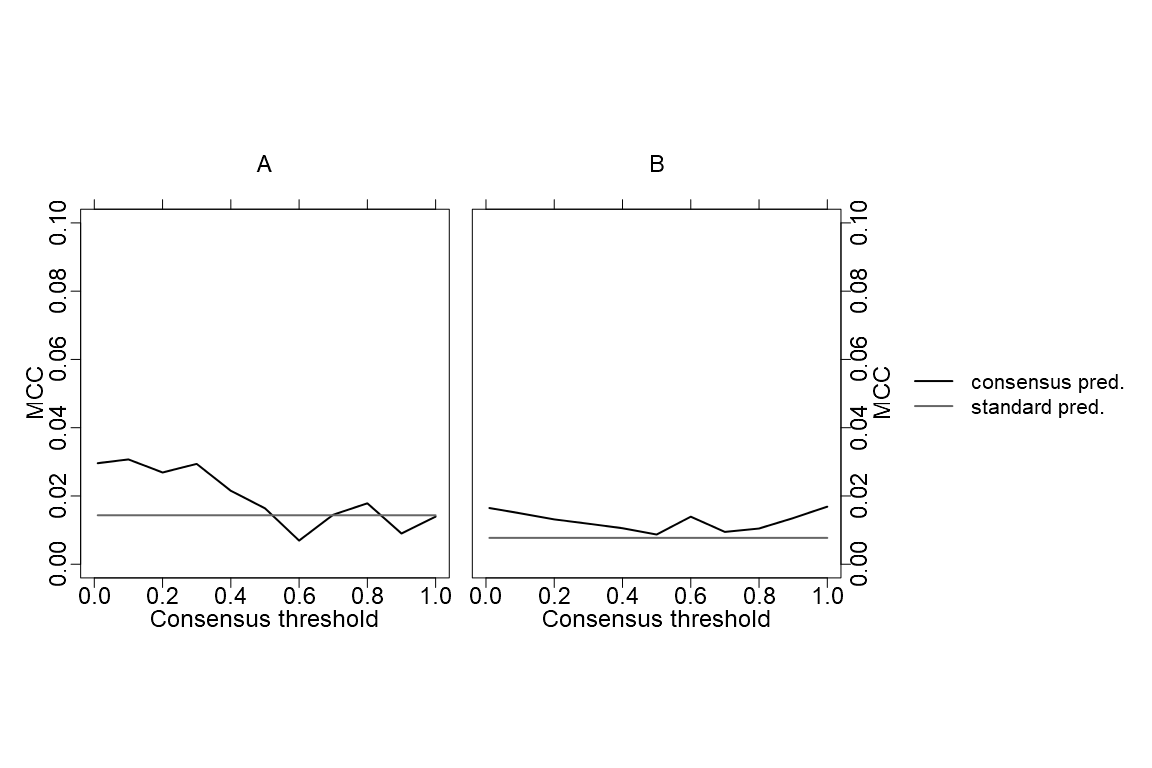


**Matthew’s Correlation Coefficient (MCC) for consensus.** MCC at different consensus thresholds. Panel A shows predictions of BindPredict-CCS, Panel B predictions of BindPredict-CC. While the MCC is at a rather low level for all methods, the consensus approach manages to outperform the standard prediction for consensus thresholds up to 0.5 in the BindPredict-CCS case, and over the whole threshold range for BindPredict-CC.
